# On the Theory and Practice of Midwifery

**Published:** 1848-05

**Authors:** 


*On the Theory and Practice of Midwifery.* By FLEETWOOD CHURCHILL, M. D., M. R. I. A., Licentiate of the College of Physicians in Ireland; Physician to the Western Lying-in Hospital; Honorary Member of the American National Institute and of the Philadelphia Medical Society. *With Notes and Additions by* ROBERT M. HUSTON, M. D., Professor of Materia Medica and General Therapeutics, and formerly of Obstetrics and the Diseases of Women and Children in the Jefferson Medical College of Philadelphia; President of the Philadelphia Medical Society, &c. &c. *Third American Edition. Revised and improved by the Author; with one hundred and twenty-eight Illustrations from Drawings by Bagg and others, engraved by* Gilbert. 8vo. pp. 525. Lea & Blanchard. Philadelphia: 1848.

Amidst the many valuable works on Obstetrics, the one before us stands prominent. It has been extensively received amongst the profession, and as extensively appreciated. Without invidious comment, it may be—will be—admitted, that no one holds a higher position, or is more deserving of being placed in the hands of the tyro, the more advanced student, or the practitioner. It is not restricted to the *ipse dixit* of the author, oracularly expressed; but contains the views of others eminent in the craft, and is thus an epitome of existing authorized and authoritative opinions. In his preface to this "*Third American Edition,*" the author states, that he has been requested by the American publishers to revise this edition, and to make such additions as the progress of science may have rendered necessary. "This," he remarks, "I have done so far as time permitted; and though I confess the work is far from being as complete as I could wish, yet I see no reason to modify or change the principles therein inculcated. An extended experience will, of course, in all such cases involve the insertion of new matter; and owing to the industry of my friend Dr. Huston, I feel satisfied that few facts of importance have been omitted. I owe a large debt of gratitude to my kind American friends, which I gladly take this opportunity of acknowledging, and also to the profession in America for the flattering reception they have given to my volumes. No reward could be more highly valued by me, nor could anything make me more anxious, by labour and study, to make my works as perfect as possible, than the knowledge that their usefulness may extend to another hemisphere."

The amiable author is not alone in his feelings of gratitude. They, who have been so much instructed by his zealous efforts in the cause of medical science, owe *him* a reciprocation of such sentiments. His works have been highly useful; and that they have been so is signally shown by the extensive sale which they have had among us. The American profession—taken as a whole—are eminently capable of appreciating true merit, and as anxious to avail themselves of the decidedly useful; and although petty jealousies and envies may, at times, prevent justice from being rendered to such as have risen far above their associates in the localities in which they reside, the body of the profession will be sure to regard them as they deserve. On the other hand, it is perfectly consistent with every day's history, that an individual, conspicuous in the restricted circle that immediately surrounds him, to an observer at a very limited distance may be a mere speck, scarcely distinguishable by telescopic agency in the nebulosity that environs him: such nebulosity may, indeed, be the only circumstance that leads the observer to a suspicion of his existence.

